# Evolution of language: An empirical study at eBay Big Data Lab

**DOI:** 10.1371/journal.pone.0189107

**Published:** 2017-12-20

**Authors:** David Bodoff, Ron Bekkerman, Julie Dai

**Affiliations:** 1 Department of Business Administration, University of Haifa, Haifa, Israel; 2 Department of Information and Knowledge Management, University of Haifa, Haifa, Israel; 3 Department of Computer Science, University of Haifa, Haifa, Israel; University of Rijeka, CROATIA

## Abstract

The evolutionary theory of language predicts that a language will tend towards fewer synonyms for a given object. We subject this and related predictions to empirical tests, using data from the eBay Big Data Lab which let us access all records of the words used by eBay vendors in their item titles, and by consumers in their searches. We find support for the predictions of the evolutionary theory of language. In particular, the mapping from object to words sharpens over time on both sides of the market, i.e. among consumers and among vendors. In addition, the word mappings used on the two sides of the market become more similar over time. Our research contributes to the literature on language evolution by reporting results of a truly unique large-scale empirical study.

## Introduction

Social and economic conventions are widespread [1, 2]. A convention is a pattern of behavior to which everyone conforms, and expects others to conform [3]. In the context of language, an object’s name is a linguistic convention—the association between an object and a word is arbitrary [4], but once it is established, it behooves everyone to use the word in the accepted way. Scientists from many fields have tried to understand how conventions form in the absence of a coordinating authority. Conventions appear to emerge in a bottom-up fashion, as a result of repeated interactions among individuals. The idea that global norms emerge from local interactions has captured the imagination of scientists working in many fields [5] and has been addressed from the perspectives of game theory [6], linguistics [7], computer science [8], logic and philosophy [3, 9], sociology [10], psychology [11], organization science [12], and other disciplines.

In spite of the widespread scientific interest in the bottom-up emergence of naming and other conventions, attempts to test the theory have been very limited. Most studies have used computer simulations [5, 13]. Empirical work is scarce, and with rare exceptions (e.g. [14]) is based on small laboratory studies. While such studies are useful for theory development, it is difficult to generalize from their results to the real world. One problem is that the size and structure of a network are known to influence how norms spread through a population [15]. Thus, while much of the interest in evolutionary theories is how they can lead to norms at a national or global scale, the existing empirical work does not remotely match the envisioned scale.

In this study, we report the results of a large-scale empirical field study on the emergence of naming conventions. The study was conducted as part of the eBay Big Data Lab project. eBay is an online marketplace with approximately 120 million active users at the time of our study. The main purpose of any marketplace is to help sellers and buyers to find one another. Online, this function is often achieved by using keywords. Vendors choose keywords with which to name or describe their item, and consumers choose keywords to search for the item they intend to purchase. In this manner, both vendors and consumers choose words for an intended item. Furthermore, the data is recorded over time. And, the data tells us which words were used (in what frequencies) for each kind of item. This is what makes the eBay data a unique basis for investigating the emergence of naming conventions, which associate words with objects.

Applied to the case of language, theories of bottom-up emergence predict that gradually over time, consensus will emerge within a population about what word to use for a given object. There are two theoretical approaches to modeling how this occurs. In an “individual learning” approach [16], individuals learn and adapt through their repeated attempts to communicate with others. By contrast, in an “evolutionary learning” approach, individuals do not necessarily change but the population as a whole “learns” by changing its composition, with an increasing proportion of people all acting in the same (successful) manner [17]. In some models, this change happens because children mimic their parents and successful communicators have more children [18]. In other models, new arrivals mimic the behavior of successful communicators [1]. Either way, the population is gradually comprised of individuals with homogeneous strategies. The specific behavior that we study here could be predicted in principle by both kinds of models—individual learning and evolutionary learning—but for a number of reasons, we adopt an evolutionary perspective. One reason is the progress made by Nowak and colleagues in showing how basic evolutionary models can be modified to account for various aspects of language evolution [19–22]. A second reason is that the individual learning framework does not naturally apply in our setting, because it would require that the same individuals repeatedly purchased the same item on eBay, and precisely in the two time windows that comprise our data; these cases would be a tiny minority of our data. For these reasons we attribute the empirical evidence as specifically supporting the evolutionary approach. In any event, the evidence shows that conventions emerge through a learning process of some kind.

One of the central predictions of an evolutionary model as it pertains to naming conventions is that, over time, consensus will emerge about what word to use for a given object. In terms of the formalism developed by Nowak and colleagues [22–24], at the outset different individuals may use different words to refer to a given object. Over time, the distribution will sharpen, as a larger proportion of the population uses the same word for that object [23]. Versions of this prediction have been tested in small laboratory studies, but to our knowledge, no evidence from real data has ever been presented in support of this most basic prediction of evolutionary theories of language.

### Prediction 1: Over time, consensus will emerge about what word to use for a given object

**Fig 1:**
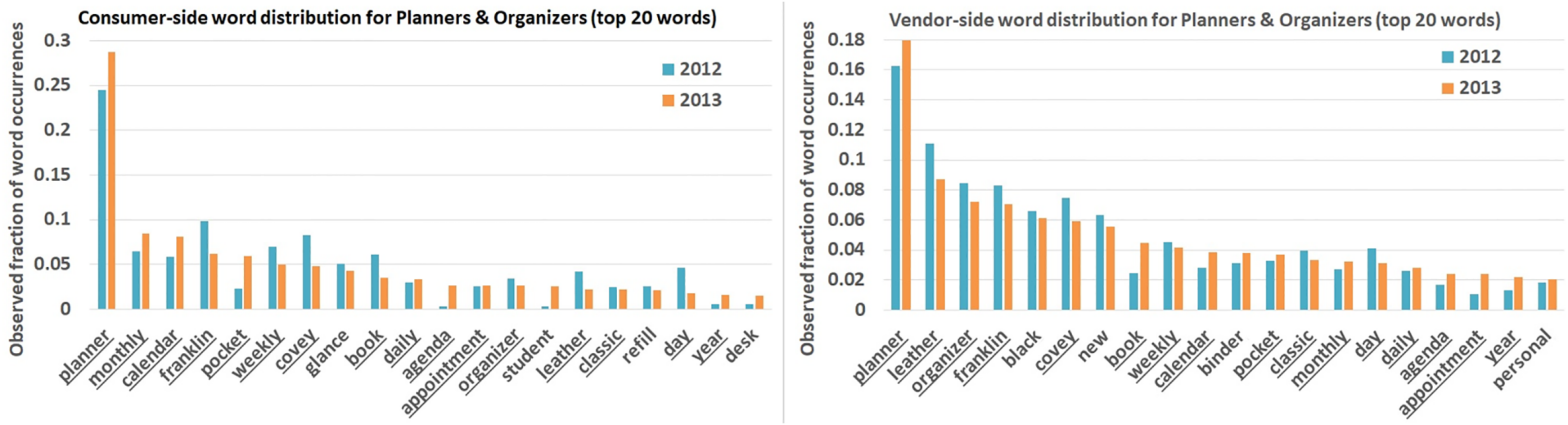
Left: Distribution of top words for Planners & Organizers category for consumers. Right: Distribution of top words for Planners & Organizers category for vendors. This example illustrates the simplest application of Predictions 1 and 2. For illustration, we consider just the leading term. On each side of the market, “planner” was the lead term in 2012. And on each side of the market, this lead term was further strengthened in 2013. In this manner, the distribution of words on each side of the market became sharper, and the entropy of the word distribution on each side of the market decreased correspondingly. Underlined are words that are in the top-20 list for *both* consumers and vendors.

Our data includes all item titles (vendor side) and search queries (consumer side) used on eBay over two 2-month periods in 2012 and 2013. To define an “object”, we used eBay’s extensive hierarchy of item categories and considered a leaf category as an object (see Materials & Methods). For example, consider eBay leaf category 3295: [Business & Industrial → Office → Office Supplies → Calendars & Planners → Planners & Organizers]. Fig 1 shows the actual consumer and vendor side word distributions for items in that category. The words on the x-axis of each figure are ordered according to their 2012 proportions. It can be seen that each of the two distributions was sharper or “peakier” in 2013 than in 2012, towards the word “planner”. This is the kind of evidence that we will seek on a large scale.

A second question regards the interaction between the two populations. On eBay and in other economic settings, there are two naturally occurring populations—consumers and vendors—that are trying to communicate with one another. The two populations may differ systematically at the outset, in terms of the words they use to refer to a given object. This difference raises interesting questions, such as how the two groups influence one another [17], and whether and which convention is expected to emerge when the two populations begin with competing word preferences [25]. These issues have received relatively little attention in the literature but they are important in economic settings.

The evolutionary theory predicts that, for the same reasons that each population’s word distribution for a given object should sharpen as discussed above, the two interacting populations should converge towards using the *same* words as one another, for the given object. This occurs because each population, wanting successful communication, has a self-interested incentive to use the terminology that it sees in use by the other population. Ultimately, this benefit drives the two populations towards one another’s term usage:

### Prediction 2: Over time, consumers’ and vendors’ word distributions for a given object will be more similar to each other

In the example of Fig 1, if we focus on the leading term for illustration, we see that not only did the consumer-side and vendor-side distributions each sharpen as discussed earlier, but the two distributions sharpened around the *same* term. Of course, in this simple example the two predictions coincide, because the same term was the leading term on both sides of the market in 2012.

The more interesting case is when the two communicating populations differ at the outset in their terminology for a given object. The evolutionary framework predicts that in this case as well, eventually the two sides should agree on a terminology, since both sides benefit from this.

**Fig 2:**
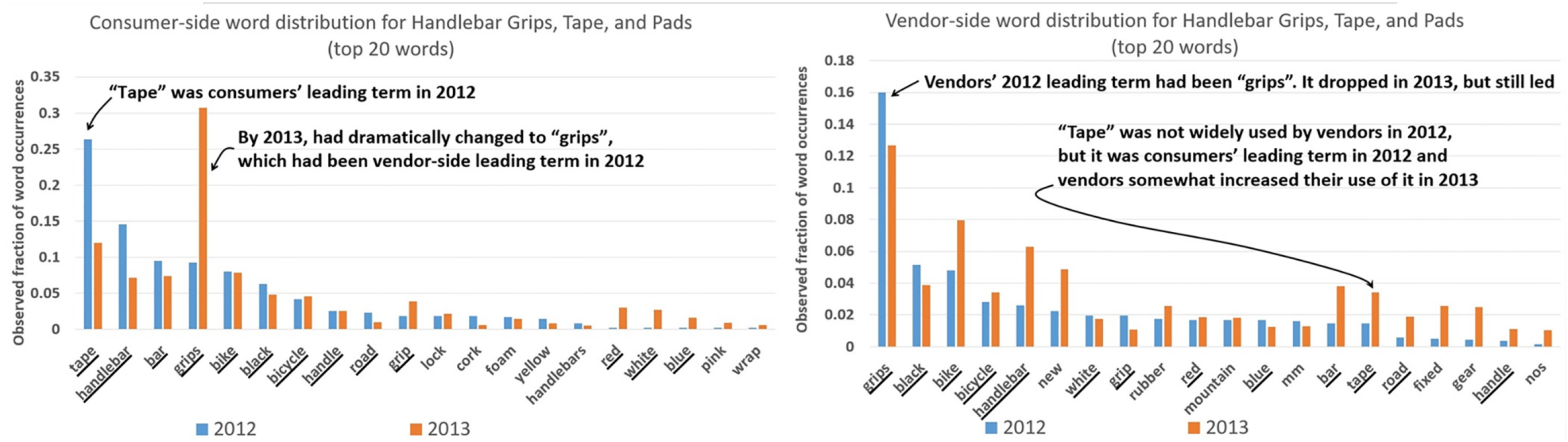
Left: Distribution of top words for Handlebar Grips for consumers. Right: Distribution of top words among vendors. Vendors in 2012 used “grips” more than “tape”, while for consumers it was the opposite. Consumers learned from vendors and by 2013 they, too, showed a strong preference for “grips”. At the same time, vendors somewhat muted their strong preference for “grips” and began using “tape” in accommodation of consumers’ 2012 word usage. In the end, because consumers made a strong accommodation and vendors made a slight one, both sides ended up favoring “grips”, and thenceforth we would expect uncomplicated mutual convergence around “grips”. Underlined are words that are in the top-20 list for *both* consumers and vendors.

This phenomenon is observed in numerous examples in the data, such as eBay category 177816: [Sporting Goods → Cycling → Bicycle Components & Parts → Handlebar Grips, Tape and Pads] as shown in Fig 2. For illustration, we focus on the leading terms on each side of the market. In 2012, the two sides of the market had opposite term preferences, with consumers using “tape” and vendors using “grips”. By 2013, consumers had dramatically accommodated vendors, adopting vendors’ use of “grips” in a swift reversal. At the same time, vendors had slightly accommodated consumers through a slight increase in their use of “tape” and reduction in the use of their lead term “grips”. The net result is that by 2013 the distributions on the two sides of the market were more similar, with both populations using “grips” as their leading term.

A third prediction explicitly reflects the hypothesized learning mechanism that is the basis for all the other predictions. The evolutionary model implies that words that were popularly used by vendors for a given object at time *t* − 1 are more likely to be used by consumers for that object at time *t*, and vice versa. This prediction directly reflects the theorized mechanism that leads to all the other results, i.e. tighter word distributions within each side of the market, and more similar word distributions between the two sides. This prediction is the most direct measure of the hypothesized dynamics themselves, and can be tested using a time-lag regression.

### Prediction 3: At the level of individual terms, consumers’ use of word *w* at time *t* will be influenced by the extent of its usage among vendors at time *t* − 1, and vice versa

To summarize, our research plan is to empirically test key predictions from the evolutionary theory of language, part of the wider body of work on the emergence of conventions. Prior empirical work on the evolution of conventions has been scarce and mostly consisted of small laboratory studies. Also, many methodological issues have never been addressed, such as how to measure the predicted narrowing of word distributions, and how to divide the world into objects (see Materials and Methods). We report empirical evidence for specific predictions regarding the emergence of naming conventions in an economic setting, as predicted by evolutionary theory. Along the way, by addressing methodological issues, we help advance the empirical study of evolutionary theories of conventions.

## Related work

Our research differs in important ways from prior literature on naming conventions. Centola and Baronchelli [2] focus most closely on the phenomenon we consider here, namely, the emergence of a commonly accepted word to use as the name for a given referent. Using a controlled experiment, they vary the size (up to 96 subjects) and structure of the network of interactions, and explore how these settings affect the formation of a naming convention for a single object. Our work differs dramatically in the naturalness, size, and other aspects of the setting, with millions of market participants interacting freely over 21 months and over twelve thousand objects. Our setting also has two distinct populations, which raises the additional questions noted above. Selten and Warglien [26] conducted laboratory studies of up to 40 subjects to explore the emergence of efficient codes, based on a fixed set of allowable letters, to refer to geometrical objects. In their experiments, each pair of subjects interacted 60 times. Our work differs again in the naturalness and scale of our field study, which also did not restrict the set of legal letters or words, or allow (force) fixed pairs of individuals to interact repeatedly. Also, the methods used in both works [2, 26] facilitate individual learning rather than evolutionary learning, since the subjects—or even the same fixed pairs of subjects—repeatedly interact. This differs from our setting where individual learning is barely relevant and evolutionary learning is the apparent mechanism through which the conventions emerge.

Other work differs from ours in other ways besides the small, controlled methods. Silvey et al. [27] study the mapping from words to objects e.g. homonymy, which is the opposite of our focus the mapping from objects to words e.g. synonymy. Still other work on evolutionary models of language regards the evolution of syntax; this is outside our scope, as is the very separate question—sometimes also called “language evolution”—of how humans evolved the physiological and cognitive capability for language [7]. Another body of work called “culturomics” [28] includes empirical studies of cultural phenomena using large textual corpora. One aspect of culturomics that is tangentially relevant to our work explores how the name for an individual event or item changed over the years, e.g. “The Great War” to “WWI”. Our work differs in that we test general naming phenomena (e.g. reduced synonymy) that are predicted by a theory of language evolution, whereas culturomics is used to describe individual, culturally significant name changes.

## Materials and methods

### Field study

We study language evolution, a phenomenon that is most meaningful when observed on a large scale in a natural setting. This attempt raised a number of methodological questions, especially: (a) how to divide the universe of items into “objects” whose word distributions are expected to sharpen; and (b) how to measure the degree to which a naming convention has emerged for a given object. In lab studies, the first of these issues is sidestepped, as the experiment implicitly pre-divides things into object stimuli. The second of these issues also does not arise in lab studies. For example, in many lab studies subjects choose from a small set of choices, and convergence is measured as the percentage of matches or other game-theoretic measures, whereas in our case—i.e. in the case of real language—it is more natural to think in terms of word distributions. We explored a number of possible approaches to addressing these methodological questions, and describe our choices here.

### Setting

The study took place at eBay Labs in Israel, as part of the eBay Big Data Labs project. The eBay Big Data Lab is an initiative of eBay Labs in which academics who wish to test theory on empirical data, or startup companies that wish to train or test their algorithms on realistic data, compete for the chance to gain access to parts of eBay’s historical data. The data is prepared for access by an eBay team. The data is sterilized to enforce privacy policies, eliminate sensitive fields, etc. eBay also provides training on how the eBay data is organized, and in the use of big data analysis tools that are available in the eBay Labs setting. The tools are provided over high-end Big Data infrastructure: a Hadoop 2.0 cluster with close to 10,000 nodes and more than 150 Petabytes of storage.

### Time periods

The time period of data that was available to us was April 2012 to December 2013. In order to avoid the special holidays period when eBay data acts differently comparing to the rest of the year, we used the following two 2-month periods as the beginning and end points of our analysis: April-May 2012 and September-October 2013.

### Objects and words

The basic idea of the analysis is to take all words used by all consumers in reference to a particular kind of object at each of the two time periods; and all words used by all vendors in reference to a particular kind of object at each of the two time periods; and derive the observed word distribution for each kind of object, at each of two time periods, for consumers and for vendors. Each such distribution could then be characterized using information-theoretic measures. On the vendor side, we have a record of all words that all vendors used to describe each and every item; and on the consumer side, we have all words that all consumers used to search for each and every item (see section Words below). A central methodological issue was how to group all the available eBay items into “objects”. This and other methodological details are described next.

#### Objects

The theory is about how the association between objects and words evolves and sharpens over time. In order to test this theory, it is necessary to define what an “object” is. This problem is assumed-away in lab or simulation studies, where the experimenter pre-divides the world into objects and where the human or simulated try to communicate about it. In real life, there is no experimenter who pre-divides the world into objects. (Indeed, a more complete theoretical approach is to simultaneously model both the process whereby speakers of the language divide items into objects, and the process whereby they give those objects names [29]). For example, in eBay, every single instance for sale of a “*Samsung Galaxy S III GT-I9300*—*32GB*—*Marble White (Unlocked) Smartphone Band*:*GSM*/*GPRS*/*EDGE 850*/*900*/*1800*/*1900 (Quadband) HSPA+ 850*/*900*/*1900*/*2100 Storage Capacity*:*32 GB Network Technology*:*GSM*/*GPRS*/*EDGE*, *HSPA+*” is a separate item, and so is every instance of every single other item offered for sale. But clearly, when the theory conceived that language speakers will converge on a name, the intention is not that they will converge on a different name for each instance of the object, or even for each slightly different item such as phone model, but that they will develop a general word such as “smartphones” for a kind of item. The question is, how can we divide the universe of eBay items into objects at the level of aggregation for which we might expect people to develop names?

We addressed this by using eBay’s metadata, which includes a hierarchical tree of categories. There are about 17,000 leaf categories. Each item for sale belongs to a single leaf category. Examples of leaf categories include “wristwatches”, “slippers”, “prayer beads”, “planners and organizers”, etc. An object in our study is defined as a leaf category.

#### Objects included in the analysis

**Fig 3:**
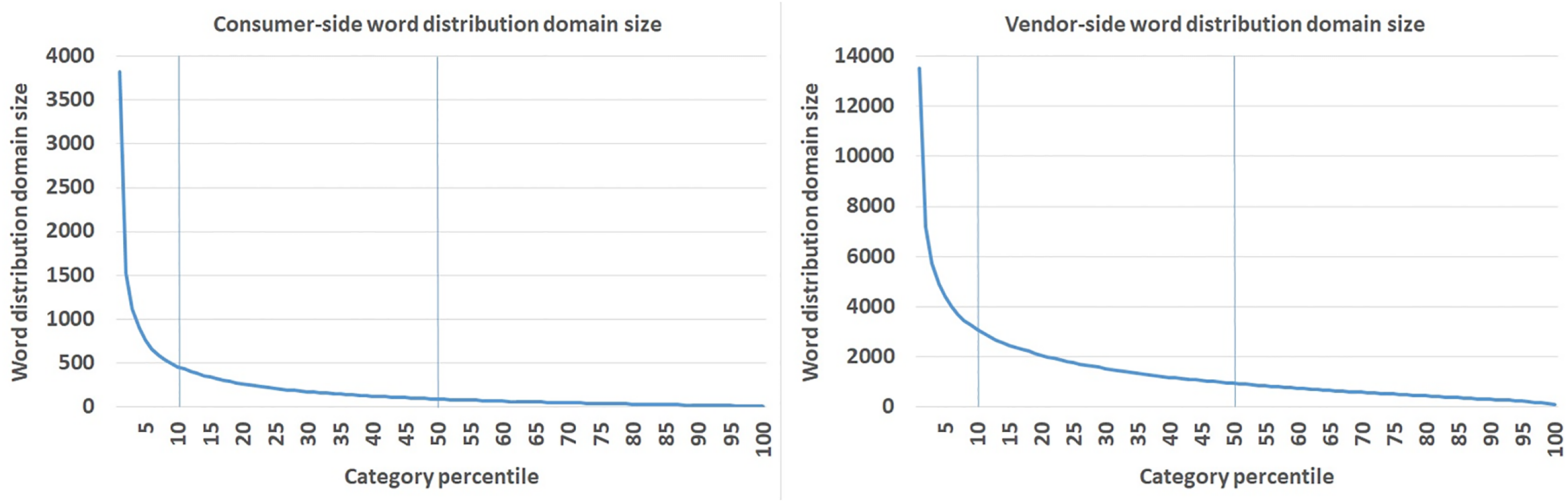
Left: Consumer-size word domain distribution sizes per category percentile. Right: Vendor-size word domain distribution sizes per category percentile. We sorted categories according to the consumer-side word domain distribution sizes and reported on top 10%, top 50%, and all 100% of 11,838 categories.

We excluded data from any category whose name had changed during the period April 2012 − October 2013. The reason is that a change in a category’s name could signify a change in the scope of products that are included, and this means that any sharpening in the word distributions could be an artifact if the category had narrowed its scope. For a similar reason, we also eliminated any category that merged with another or that gained or lost a “sibling” in the category hierarchy, because any of these cases could mean that the scope of what is included in that category could have changed, which ruins our effort to track changes in word distributions for a “given” kind of object. Approximately 2,000 categories were excluded for these reasons. Next, we wanted to be aware how much data we had for each category, in order to put most faith in categories with the most data. For each category, we calculated the number of unique words on the consumer side, as used in search queries in 2012 and 2013 (taken as a union). We also calculated the number of unique words on the vendor side, as used in item titles in 2012 and 2013 (union as well). Fig 3 shows those numbers per category percentile. As we can see, the top 10% (1,184) of categories are dramatically larger than the rest of the categories. We performed our analyses on the top 10% categories. For consistency, we repeated them on the top 50% of categories and on the total 100% set of 11,838 categories.

#### Words

Each item on-sale at eBay has a title. Our analysis included item titles on the USA website, and only those items that sold at least 1 unit during each of two 2-month time periods. For the vendor side, we collected all word-instances used in all item titles. Unlike the vendor side where we know with certainty what item the vendor is describing, on the consumer side, we need to make a simplifying assumption in order to know what item the consumer was searching for. The assumption we made is that if a consumer session culminated in a purchase, then we assume that is the kind of item he/she was looking for. We consider only those consumer sessions that ended in a purchase, and we associate any search terms they used during that session, with that item. This simplifying assumption is reasonable for eBay where it is typical that a session culminates in the purchase of a single item if any, rather than with a cartload of different items. All our analyses worked with single words, not phrases. We stripped out 300 stopwords—pronouns, propositions, determiners, modal and auxiliary verbs [30]—and all non-dictionary words from the data. We used the online Moby dictionary of around 350,000 words. Table 1 shows descriptive statistics of our data.

**Table 1:** Data descriptives.

Period	Market side	Search queries (Consumers) or item titles (Vendors)	Unique words	Unique words after removing stopwords and non-dictionary words	Tokens after removing stopwords and non-dictionary words
*Apr*/*May* 2012	*Consumers*	9,927,028	218,460	52,958	13,263,934
	*Vendors*	43,144,860	2,948,058	104,285	282,536,498
*Sep*/*Oct* 2013	*Consumers*	12,250,818	288,665	58,218	19,266,735
	*Vendors*	52,054,168	3,362,990	108,851	363,034,559

### Measures of distribution sharpening and similarity (Predictions 1-2)

The first prediction requires a test of whether a word distribution for a given object has sharpened. A good candidate measure of the (non-)sharpness of a distribution is the Shannon entropy of the observed (multinomial) distribution of word frequencies. Assuming an object has an observed domain of words *W*, the Shannon entropy is defined as *H*(*W*) = −∑_*w*_ *p*(*w*)log(*p*(*w*)). However, a methodological issue arises because the size of the two domains—i.e. the cardinality of the set of words—is not identical in the two periods, and in addition, the sample size grows because use of eBay Marketplace grew as a whole, and both these differences affect the expected entropy of the observed distribution. For example, if the underlying true word distribution (unobserved) is actually identical but just the sample size grew, this would lead to a higher expected entropy of the observed word sample [31]. This artifact would actually make the predicted finding of reduced entropy even stronger. Nevertheless, to allow a meaningful comparison of the two observed distributions’ entropies, for each time period we calculate its empirical entropy relative to that of a uniform distribution of the same sample size for a specific object. For each object, we calculate the relative entropy for each time period, and determine if it increases or decreases.

Prediction 2 requires a measure of the (dis-)similarity between two distributions *W*_1_ and *W*_2_. For this, we use the Jensen-Shannon (JS) divergence:

$$\begin{array}{c} JS\left(W_{1}∥W_{2}\right)=\frac{1}{2}\sum_{w}p_{1}\left(w\right)\log\frac{p_{1}\left(w\right)}{p^{*}\left(w\right)}+\frac{1}{2}\sum_{w}p_{2}\left(w\right)\log\frac{p_{2}\left(w\right)}{p^{*}\left(w\right)}, \end{array}$$

where *p*_*i*_(*w*) is the probability of word w in distribution *W*_*i*_ and $p^{*}\left(w\right)=\frac{1}{2}\left(p_{1}\left(w\right)+p_{2}\left(w\right)\right)$.

### Measures of word-level learning (Prediction 3)

Prediction 3 is tested using regression. We consider the vendor side data (for reasons explained below, the data did not allow a clean analysis of the comparable effects on the consumer side). Let *V*_*iw*,*t*_ denote the proportion of occurrences of word *w* among all word occurrences in all item titles for object *i* at time *t*. Similarly, *C*_*iw*,*t*_ denotes the proportion of occurrences of word *w* at time *t* among consumer searches for that object i.e. items in that category. We divide the analysis into two parts, one logistic regression to predict zero from non-zero values of *V*_*iw*,*t*_, and another linear regression to predict variance among the non-zero values. This split is necessary because of the many zero values in the dependent variable, which violate the normality assumptions of linear regression.

We define a binary outcome as follows:

$$\begin{array}{c} V_{iw,t}^{B}=\{\begin{array}{cc} 1, & \text{if}\;V_{iw,t}>0\\ 0, & \text{otherwise}. \end{array} \end{array}$$

We thus have two regression equations for the vendor side of the market:

$$\begin{array}{c} p(V_{iw,t}^{B}=1)=\frac{1}{1+e^{-(\beta_{0}+\beta_{1}V_{iw,t-1}+\beta_{2}C_{iw,t-1})}}, \end{array}$$ (1)

$$\begin{array}{c} V_{iw,t}=\beta_{0}+\beta_{3}V_{iw,t-1}+\beta_{4}C_{iw,t-1}+\epsilon_{iw,t},\;\text{for}\;V_{iw,t}>0. \end{array}$$ (2)

The predictors of interest are *β*_2_ and *β*_4_ that represent the theorized cross-market lagged influence. Besides testing to see if those coefficients are statistically significant, each of the two models is compared against a baseline that omits the cross-market influence.

The data did not allow a comparable test on the consumer side. The consumer side model would regress *C*_*iw*,*t*_ on *C*_*iw*,*t* − 1_ and *V*_*iw*,*t* − 1_. The problem is that on the consumer side, we only know to associate a search with an item when the search ends in a purchase of that item. But a search will only succeed in finding an item if the vendor used matching words. Thus, a consumer search is likely to be included in the data only if *C*_*iw*,*t*_ had words found in *V*_*iw*,*t*_. Since *V*_*iw*,*t*_ and *V*_*iw*,*t* − 1_ are strongly correlated, *C*_*iw*,*t*_ will be strongly correlated with *V*_*iw*,*t* − 1_ as an artifact, not necessarily because consumers are lagged-learning vendors’ terminology. Indeed, a regression showed a very strong effect of *V*_*iw*,*t* − 1_ on *C*_*iw*,*t*_. But the data did not allow us to untangle the learning effect from the artifact.

## Aggregate results

Prediction 1 was that per-object word distributions will sharpen over time. Working with the data and the proposed *relative entropy* measure (see Materials and Methods), we tabulate the number of per-object distributions whose entropy decreases versus increases over time. Table 2 shows the number of objects that exhibit a decrease in entropy over time, as predicted, versus the number that exhibited an increase. The analysis is repeated three times, using the most frequently searched top 10% of objects, the top 50%, and the full set of 11,838 objects. As shown in the table, on each side of the market, for all sub-samples of the data, entropy went down for more than half the number of objects. Using a binomial test, the proportion was statistically larger than a random half in all cases.

**Table 2:** Results for Prediction 1: Entropy changes, relative to uniform.

Market side	Cutoff	Number of objects for which entropy rose	Number of objects for which entropy fell	Statistical significance (one-tailed)
*Vendors*	*Top* 10% *objects*	469	715	*p* < .0001
	*Top* 50% *objects*	2416	3503	*p* < .0001
	100% *objects*	4948	6890	*p* < .0001
*Consumers*	*Top* 10% *objects*	489	695	*p* < .0001
	*Top* 50% *objects*	2556	3363	*p* < .0001
	100% *objects*	5050	6788	*p* < .0001

Prediction 2 was that the two sides’ (consumers and vendors) per-object word distributions will become more similar to one another over time. Table 3 shows the numbers of objects for which JS divergence increased (i.e. word distributions became more different) versus decreased (i.e. word distributions became more similar). As shown, for all sub-samples of the data, JS divergence went down for more than a half of the objects. Using a binomial test, the proportion was statistically larger than a random half in all cases.

**Table 3:** Results for Prediction 2: Changes in JS divergence in the two sides’ word distributions.

Cutoff	Number of objects for which JS divergence rose	Number of objects for which JS divergence fell	Statistical significance (one-tailed)
*Top* 10% *objects*	278	906	*p* < .0001
*Top* 50% *objects*	1827	4092	*p* < .0001
100% *objects*	4250	7588	*p* < .0001

**Fig 4:**
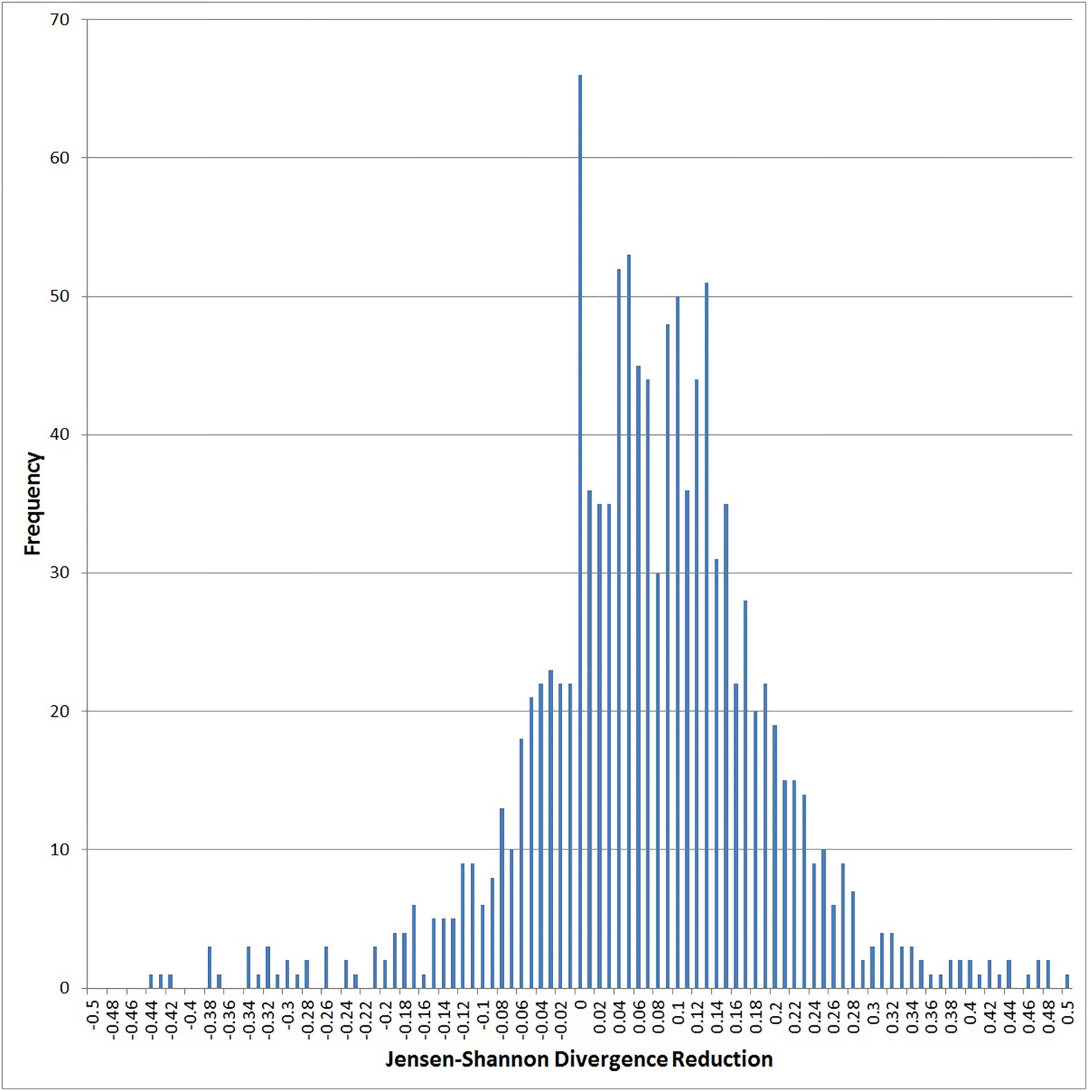
Further analysis of Prediction 2: Percent reduction in JS divergence, for largest 1,184 objects.

Fig 4 shows a histogram of the magnitude of this effect, as a percentage reduction in JS divergence per object between the two periods. The median change was 7% reduction. We view this as a very meaningful magnitude of result, considering that the period is just over one year.

**Fig 5:**
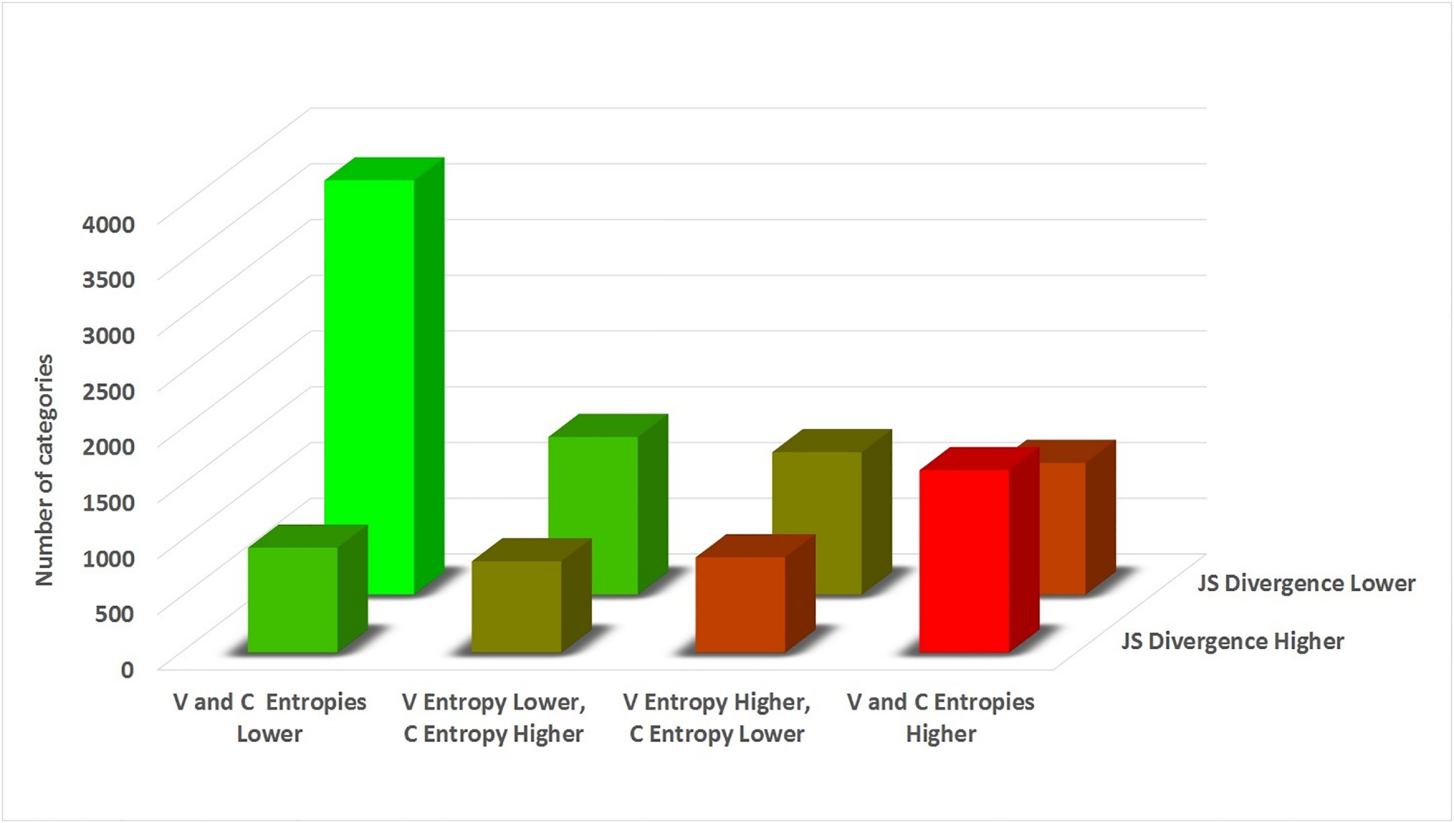
Further analysis of Predictions 1-2: The pattern of entropy / JS divergence increase/decrease between Vendors (V) and Consumers (C), for all objects.

Fig 5 shows the main results even more clearly. The figure analyzes the two main results in tandem. It shows the number of objects that exhibit a rise/fall in consumer-side entropy, vendor-side entropy, and JS divergence between the two sides. The main results of our work are especially clear in this analysis. By far, the most common result is a reduction in JS divergence combined with a reduction in both sides’ entropies. This is the classic case that is predicted by the theory, wherein each side converges on specific terms (reduced entropy), and both sides converge on the *same* term (reduced JS divergence). In addition, by showing that the two sides usually sharpen their word distributions when gaining agreement with the other side, the analysis suggests that the two sides are sharpening their word distributions *in order* to gain sharp agreement with the other side. This is exactly the logic of the evolutionary process.

Prediction 3 directly captures the theorized learning mechanism that is responsible for other results. It is tested by regression equations. The predictors of interest are *β*_2_ and *β*_4_ that capture the cross-market lagged influence. Each of the two models is compared against a baseline that omits the cross-market influence.

Table 4 shows results. For the logistic regression (Eq 1), the hypothesized model is not only a better predictor with higher R-square and lower AIC values, it is really the only viable model; the baseline model does not fit the data at all—the ROC value is below the commonly accepted minimum value of.7, the ROC graph falls under the diagonal, and the negative value on coefficient *β*_1_ cannot be meaningfully interpreted. These problems are all remedied in the hypothesized model of Eq 1 (the negative lagged coefficient becomes reasonable when combined with the positive effect of the cross-market predictor *C*_*iw*,*t* − 1_). This improvement provides evidence for the hypothesized model. In the linear regression (Eq 2) as well, the cross-market predictor is a better fit with a small effect, though it is not as strong as the improvement seen in the logistic part. Based on all the above, we conclude that there is direct evidence for the hypothesized cross-market learning.

**Table 4:** Results for Prediction 3 regressions.

		Model Coefficients	R-square (ROC for logistic), Model Fit
*Logistic Part*	*Vendor Logistic Model* (Eq 1)	*β*_1_ = −0.25 (*p* < .0001)	R-sq = .70
		*β*_**2**_ = **0.2** (**p** < .**0001**)	AIC = 19154802
	*Vendor Logistic Baseline*	*β*_1_ = −0.21 (*p* < .0001)	R-sq = .66
			AIC = 19596958
*Linear Part*	*Vendor Model* (Eq 2) *for non-zero values of V*_*iw*,*t*_	*β*_3_ = 0.32 (*p* < .0001)	R-sq = .26
		*β*_**4**_ = **0.08** (**p** < .**0001**)	AIC = 14548730
	*Vendor Baseline for non-zero values of V*_*iw*,*t*_	*β*_3_ = 0.34 (*p* < .0001)	R-sq = .25
			AIC = 14648524

We summarize our results as follows:

Result 1: Both consumer and vendor distributions show a sharpening over time of the word distributions per object.

Result 2: Consumers’ and vendors’ per-object word distributions become more similar to one another over time.

Result 3: There is direct evidence of the theorized mechanism.

## Additional analyses

Besides the absorbing-state predictions that the two sides will converge separately (Prediction 1) and together (Prediction 2), the evolutionary framework makes specific predictions about what the path to that result will look like. Such predictions highlight the ability of the evolutionary framework to offer insights about the process itself. In this section, we investigate one such prediction.

As noted above, one of the more interesting cases arises when the two interacting populations initially used different words to refer to an object. In such cases, the evolutionary process can be conceived as having two stages. In a first stage, as the two populations evolve towards each other, the result can actually be a flattening of one or both distributions. This happens because they began with different word preferences, so the rational attempt to adopt the other side’s terminology leaves them both “in the middle” between the various word tendencies. At some point, a tipping point is reached and one word or another begins to stand out. Then the second stage occurs, in which the two sides each sharpens its word usage around that agreed-upon term, as in Prediction 1.

To motivate Predictions 1–3, we relied on a general synopsis of the literature, but the intuition behind this additional analysis is perhaps clearer if we present a simple evolutionary formalism. Consider a case with the two communicating populations using words, *A* and *B* to refer to a given object. A system of replicator equations that we present for illustration are derived from a coordination game, in which a consumer and a vendor both receive a positive payoff if they both choose the same keyword, i.e. both choose A or both choose B. Assuming a payoff of 1, a consumer’s expected payoff from choosing A is just the proportion of vendors that choose A. The deterministic replicator equations that are derived from this coordination game are:

$$\begin{array}{c} \Delta C_{A}\left(t\right)=C_{A}\left(t\right)[V_{A}\left(t\right)-\left(C_{A}\left(t\right)*V_{A}\left(t\right)+\left(1-C_{A}\left(t\right)\right)*\left(1-V_{A}\left(t\right)\right)\right)] \end{array}$$ (3)

$$\begin{array}{c} \Delta V_{A}\left(t\right)=V_{A}\left(t\right)[C_{A}\left(t\right)-\left(V_{A}\left(t\right)*C_{A}\left(t\right)+\left(1-V_{A}\left(t\right)\right)*\left(1-C_{A}\left(t\right)\right)\right)], \end{array}$$ (4)

where Δ*C*_*A*_(*t*) is the change in the proportion of consumers who choose *A* following time *t*, *C*_*A*_(*t*) is the proportion of consumers who chose *A* at time *t*, and *V*_*A*_(*t*) is the proportion of vendors who chose *A* at time *t*. The meaning of the first *V*_*A*_(*t*) in the consumer equation is that the rate of growth at time *t* in the percentage of consumers using *A* is an increasing function of the proportion of vendors that use term *A* at that time; similarly for vendors. Since the proportion of consumers that choose *B* is just 1 minus the proportion of those choosing *A* (and similarly for vendors), the equations for *B* are left as implicit.

**Fig 6:**
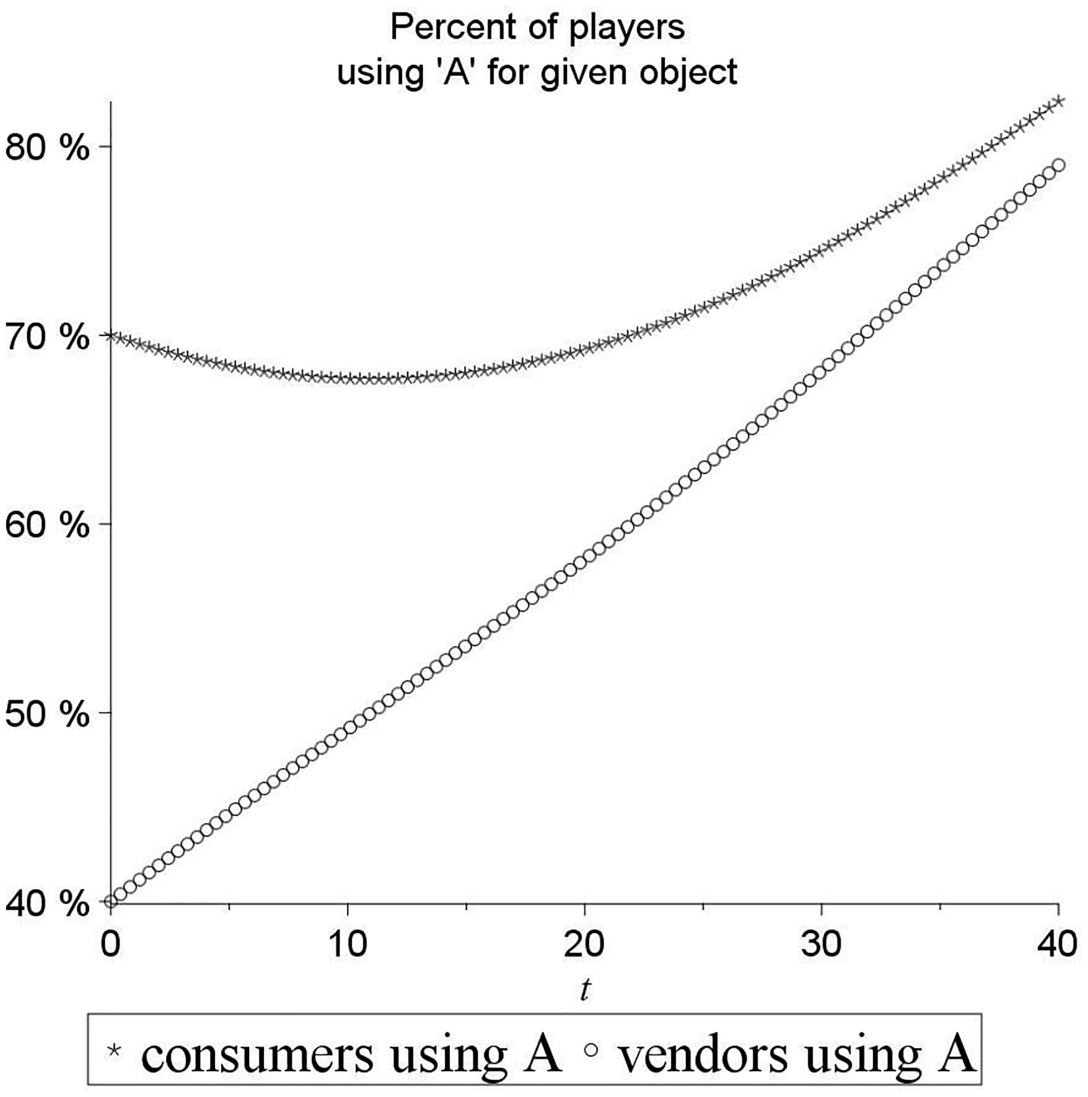
Illustration of word distribution flattening before convergence. Consumers lower the strength of their initial preference for *A*, while vendors lower the strength of their initial preference for *B*, i.e. increase their acceptance of *A*; this makes both sides closer to a 50-50 uniform split than they began, as they try to accommodate one another.

Fig 6 shows what happens in this system when initial conditions are set such that 70% of consumers use term *A* while 60% of vendors use term *B* at the outset, i.e. they conflict over the leading term. Note that this figure is not a depiction of our eBay data, but rather an illustration that conveys the intuition of the dynamic system. It is a path diagram that shows the values over time (x-axis) of the fractions of vendors and consumers using term *A* and not *B* for the given object (y-axis). The figure shows that both sides of the market first move closer to a 50-50 split, i.e. both word distributions flatten instead of sharpen, with consumers *decreasing* their use of *A* to be closer to 50% and vendors *increasing* their use of *A* to be closer to 50%. Only once a tipping point is reached, do both sides sharpen around term *A*. This simple formalism shows why the sharpening of the two sides’ distributions over a given time period, as in Prediction 1, is less likely to be observed for objects regarding which the two sides begin in terminological conflict, and more likely for objects regarding which the two sides were already in agreement over a leading term.

**Fig 7:**
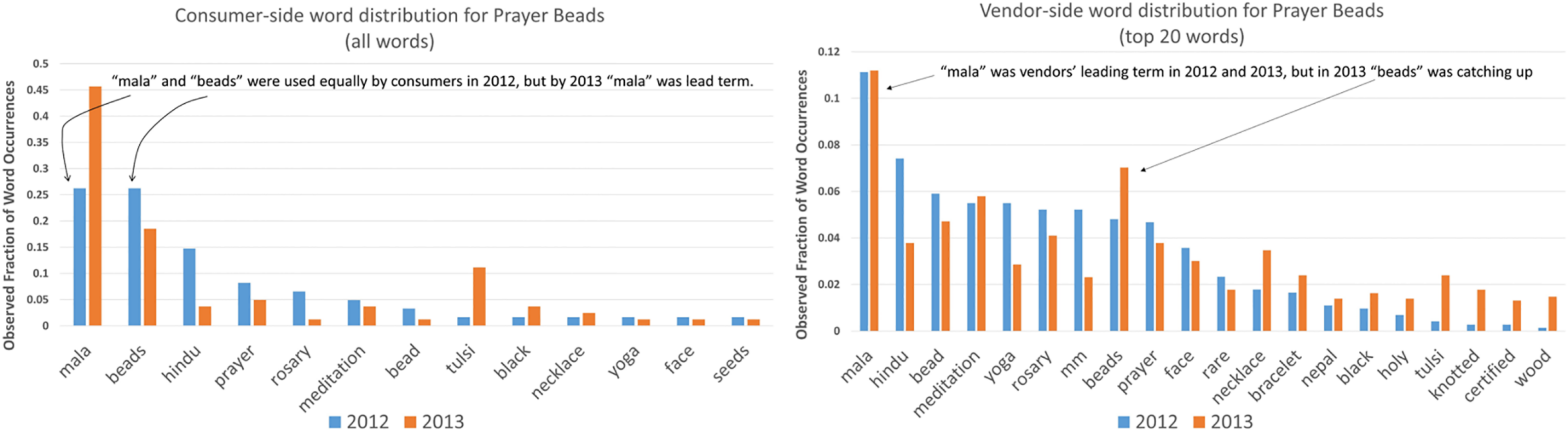
Left: Distribution of top words for Prayer Beads category for consumers. Right: Distribution of top words for Prayer Beads category for vendors. Vendors in 2012 used “mala” more than “beads”, while consumers used both equally. Consumers learned from vendors and by 2013 they showed a preference for “mala”. This change represents a sharpening of their word distribution, compared with their initial equal division between “mala” and “beads”. The result was a decrease in entropy on the consumer side. On the vendor side, the opposite effect was observed. In 2012, vendors had had a clear preference for “mala” over “beads”. By 2013, learning from consumers, vendors had muted their relative preference for “mala” by increasing their use of “beads”. The result of this flattening of the distribution was an increase in entropy on the vendor side.

This phenomenon is observed in numerous examples in the data, such as eBay category 165685: [Collectibles → Religion & Spirituality → Hinduism → Prayer Beads]. Focusing on the leading terms on each side of the market, vendors use the word “mala”, while consumers are divided between “mala” and “beads”. Vendors attempt to accommodate consumers’ word preference, and this attempt at accommodation results in their diluting their initial preference for “mala”. At the same time, consumers’ attempt to accommodate vendors’ word preference leads to consumers’ strengthening their use of “mala”, breaking the tie between the two words. This is exactly what we see, as shown in Fig 7. Vendors’ lessen the strength of their original preference for “mala” and the distribution entropy rises, while consumers adopt a preference for “mala” instead of their indifference, and the distribution entropy falls.

To directly test for this two-stage dynamic in the aggregate data, it would be necessary to examine each case (i.e. category) where the two sides begin with different words, and track the evolution, first to see a flattening, and then, after they agree on the lead term, to see a mutual sharpening. But this approach is not workable on an aggregate scale, and does not provide us with a test statistic. Instead, we run a cross-sectional test that captures the same effect. To do this, we take advantage of the fact that some objects begin with agreement over the lead term at the outset, and some with disagreement. We predict that the sharpening of distributions (as of Prediction 1) is more likely to be observed over a given period, for the objects that began in agreement about the leading term. The reason is that for the other categories, a flattening may be observed (if the time lapse is too large, we may “miss” that first stage, but still we will catch it sometimes).

### Prediction 4: The sharpening of distributions (as in Prediction 1) will be more likely regarding objects for which the two sides agreed at the outset on the leading term

Working with the aggregate data, we compared objects for which the two sides had the same leading term already in 2012, against those for which they conflicted over the leading term. For each of these two sets, we compared the percentage of objects whose entropies decreased versus increased while the JS divergence between the two sides decreased. We expected and found that in the set of objects for which the starting point had them in conflict, we were more likely to see entropy increases, i.e. the telltale pattern of flattening from accommodation. Results for the top 10% of objects are shown in Table 5. A chi-square test is significant. However, the effect becomes borderline significant when working with the top 50% objects and insignificant when working with 100% objects, probably because of the smaller number of keywords in those objects.

**Table 5:** Flattening effect. Each cell contains number of categories meeting those conditions.

	JS divergence decreased, both entropies decreased (“mutual convergence”)	JS divergence decreased, both entropies increased (“accommodation through flattening”)	Statistical significance (chi-square)
*Number of objects with the same lead term for vendors and consumers in* 2012	297	105	${\tilde{\chi }}^{2}\left(2,N=640\right)=7.75$, *p* < .01
*Number of objects with two different lead terms for vendors and consumers in* 2012	151	87	

This very specific pattern strengthens our interpretation of the main results, as representing evidence of an evolutionary process in the formation of naming conventions.

## Summary

The evolutionary theory of language is part of a larger body of research on the emergence of conventions. Applied to an economic setting with buyers and sellers, it predicts that the two populations will gradually learn to use the same words for the same items. We find evidence for all the theoretically predicted aspects of evolution and convergence, even within a period of 15 months. To our knowledge, this is the first reported evidence from a natural setting in support of the evolutionary theory of language.

The analyses provide mutually reinforcing evidence in support of the theory. The sharpening distributions is a classically predicted outcome according to evolutionary theories of language. The other propositions all add support for the theorized mechanism. The data show that the two distributions are not only sharpening but becoming more similar across the two sides of the market, i.e. sharpening around the same words, and that these two effects coincide. And, the cross-market learning mechanism can be directly measured at the level of individual words. Taken all together, we view the results as strong evidence of the theorized process as well as outcomes.

In addition to the results themselves, we also hope to have facilitated further empirical studies in realistic settings. Existing theory is fairly stylized, and existing empirical work was also largely limited to highly controlled settings. This tradition left open many methodological questions regarding how to operationalize and test the theory’s predictions in a real setting. For example, in a real setting, how is one to segment items into “objects”? How should we measure a sharpening in the distribution? And so on. We have suggested ways to address these questions, thereby helping to bridge the gap between existing stylized models and future empirical studies.

## References

[1] Young HP. The economics of convention. The Journal of Economic Perspectives. 1996;10(2):105–122. doi: 10.1257/jep.10.2.105

[2] Centola D, Baronchelli A. The spontaneous emergence of conventions: An experimental study of cultural evolution. Proceedings of the National Academy of Sciences. 2015;112(7):1989–1994. doi: 10.1073/pnas.1418838112 10.1073/pnas.1418838112 PMC4343158 25646462

[3] Lewis DK. Convention: A Philosophical study. Harvard University Press; 1969.

[4] Fromkin V, Rodman R, Hyams N. An introduction to language. Cengage Learning; 2010.

[5] Christiansen MH, Kirby S. Language evolution: Consensus and controversies. Trends in cognitive sciences. 2003;7(7):300–307. doi: 10.1016/S1364-6613(03)00136-0 12860188 10.1016/s1364-6613(03)00136-0

[6] Cubitt RP, Sugden R. Common Knowledge, Salience and Convention: A Reconstruction of David Lewis’ Game Theory. Economics and Philosophy. 2003;19(02):175–210. doi: 10.1017/S0266267103001123

[7] Bickerton D. Language evolution: A brief guide for linguists. Lingua. 2007;117(3):510–526. doi: 10.1016/j.lingua.2005.02.006

[8] Steels L. Evolving grounded communication for robots. Trends in cognitive sciences. 2003;7(7):308–312. doi: 10.1016/S1364-6613(03)00129-3 12860189 10.1016/s1364-6613(03)00129-3

[9] Wittgenstein L. Philosophical Investigations. Oxford; 1953.

[10] Biggart NW, Beamish TD. The economic sociology of conventions: Habit, custom, practice, and routine in market order. Annual review of Sociology. 2003;29(1):443–464. doi: 10.1146/annurev.soc.29.010202.100051

[11] Schelling TC. The strategy of conflict. Harvard University Press; 1980.

[12] Mauws MK, Phillips N. Crossroads understanding language games. Organization Science. 1995;6(3):322–334. doi: 10.1287/orsc.6.3.322

[13] Briscoe T. Linguistic evolution through language acquisition. Cambridge University Press; 2002.

[14] Lieberman E, Michel JB, Jackson J, Tang T, Nowak MA. Quantifying the evolutionary dynamics of language. Nature. 2007;449(7163):713–716. doi: 10.1038/nature06137 17928859 10.1038/nature06137 PMC2460562

[15] Castellano C, Fortunato S, Loreto V. Statistical physics of social dynamics. Reviews of modern physics. 2009;81(2):591–646. doi: 10.1103/RevModPhys.81.591

[16] Steels L, Kaplan F. Aibo’s first words: The social learning of language and meaning. Evolution of communication. 2000;4(1):3–32.

[17] Axelrod R. An evolutionary approach to norms. American political science review. 1986;80(04):1095–1111. doi: 10.1017/S0003055400185016

[18] Van Huyck J, Battalio R, Mathur S, Van Huyck P, Ortmann A. On the origin of convention: Evidence from symmetric bargaining games. International Journal of Game Theory. 1995;24(2):187–212. doi: 10.1007/BF01240042

[19] Nowak MA, Komarova NL, Niyogi P. Evolution of universal grammar. Science. 2001;291(5501):114–118. doi: 10.1126/science.291.5501.114 11141560 10.1126/science.291.5501.114

[20] Nowak MA, Komarova NL, Niyogi P. Computational and evolutionary aspects of language. Nature. 2002;417(6889):611–617. doi: 10.1038/nature00771 12050656 10.1038/nature00771

[21] Komarova NL, Niyogi P, Nowak MA. The evolutionary dynamics of grammar acquisition. Journal of theoretical biology. 2001;209(1):43–59. doi: 10.1006/jtbi.2000.2240 11237569 10.1006/jtbi.2000.2240

[22] Nowak MA, Komarova NL. Towards an evolutionary theory of language. Trends in cognitive sciences. 2001;5(7):288–295. doi: 10.1016/S1364-6613(00)01683-1 11425617 10.1016/s1364-6613(00)01683-1

[23] Nowak MA, Plotkin JB, Krakauer DC. The evolutionary language game. Journal of Theoretical Biology. 1999;200(2):147–162. doi: 10.1006/jtbi.1999.0981 10504282 10.1006/jtbi.1999.0981

[24] Nowak MA, Krakauer DC. The evolution of language. Proceedings of the National Academy of Sciences. 1999;96(14):8028–8033. doi: 10.1073/pnas.96.14.8028 10.1073/pnas.96.14.8028 PMC22182 10393942

[25] Helbing D, Yu W, Opp KD, Rauhut H. Conditions for the emergence of shared norms in populations with incompatible preferences. Plos One. 2014;9(8). doi: 10.1371/journal.pone.0104207 10.1371/journal.pone.0104207 PMC4148260 25166137

[26] Selten R, Warglien M. The emergence of simple languages in an experimental coordination game. Proceedings of the National Academy of Sciences. 2007;104(18):7361–7366. doi: 10.1073/pnas.0702077104 10.1073/pnas.0702077104 PMC1863456 17449635

[27] Silvey C, Kirby S, Smith K. Word meanings evolve to selectively preserve distinctions on salient dimensions. Cognitive Science. 2015;39(1):212–226. doi: 10.1111/cogs.12150 25066300 10.1111/cogs.12150

[28] Michel JB, Shen YK, Presser A, Veres A, Gray MK, Pickett JP, et al Quantitative analysis of culture using millions of digitized books. Science. 2011;331(6014):176–182. doi: 10.1126/science.1199644 21163965 10.1126/science.1199644 PMC3279742

[29] Yu C. The emergence of links between lexical acquisition and object categorization: A computational study. Connection science. 2005;17(3–4):381–397. doi: 10.1080/09540090500281554

[30] Bekkerman R, Gavish M. High-precision phrase-based document classification on a modern scale. In: Proceedings of the 17th ACM SIGKDD international conference on Knowledge discovery and data mining; 2011. p. 231–239.

[31] Roy B. Bounds on the expected entropy and KL-divergence of sampled multinomial distributions. Unpublished manuscript, MIT; 2011.

